# An Overview of the Effects of Social Media Use on Youth and Adolescent Mental Health

**DOI:** 10.1192/j.eurpsy.2026.11177

**Published:** 2026-07-31

**Authors:** N. Agyapong-Opoku, F. Agyapong-Opoku, B. Agyapong, A. Greenshaw

**Affiliations:** 1 University of Ghana, Accra, Ghana; 2 Deparment of Psychiatry, University of Alberta, Edmonton, Canada; 3 School of Medicine, University of Galway, Galway, Ireland

## Abstract

**Introduction:**

The rapid increase in social media use among adolescents has raised growing concerns about its impact on mental health. Despite extensive research, findings remain inconsistent, with evidence suggesting both negative and positive effects. Understanding this complex relationship is crucial to guide interventions and inform public health strategies.

**Objectives:**

This scoping review synthesizes current evidence on the effects of social media use on adolescent mental health, identifies consistent patterns and discrepancies across studies, highlights research gaps, and proposes directions for future investigation.

**Methods:**

A scoping review was conducted following Arksey and O’Malley’s five-stage framework. Searches were performed in PubMed, MEDLINE, Web of Science, and Scopus for articles published between July 2020 and July 2024. Eligible studies included systematic reviews, umbrella reviews, narrative reviews, and meta-analyses in English that examined social media use and mental health outcomes among adolescents. Of 1,005 articles identified, 43 met the inclusion criteria. Data were extracted on study characteristics, outcomes, and thematic findings.

**Results:**

Most studies reported associations between social media use and adverse mental health outcomes, particularly depression and anxiety. Problematic or excessive use and passive consumption were most consistently linked to negative effects, whereas active engagement was sometimes associated with benefits such as increased social support and reduced loneliness. During the COVID-19 pandemic, results were mixed, reflecting both the protective and detrimental aspects of online connectivity.

**Conclusions:**

The impact of social media on adolescent mental health is complex and context dependent. Effects vary according to usage patterns, individual vulnerabilities, and environmental influences. Future research should prioritize longitudinal designs to clarify causal pathways and inform the development of targeted interventions. Enhancing digital literacy and creating supportive online environments remain essential for optimizing the benefits of social media while minimizing its risks.

**Disclosure of Interest:**

None Declared

